# Factors associated with influenza vaccination among healthcare workers in acute care hospitals in Canada

**DOI:** 10.1111/irv.12545

**Published:** 2018-03-02

**Authors:** Hadia Hussain, Allison McGeer, Shelly McNeil, Kevin Katz, Mark Loeb, Andrew Simor, Jeff Powis, Joanne Langley, Matthew Muller, The Canadian Health Care Worker Study Group, Brenda L. Coleman

**Affiliations:** ^1^ Mount Sinai Hospital Toronto ON Canada; ^2^ University of Toronto Toronto ON Canada; ^3^ Queen Elizabeth II Health Sciences Centre Halifax NS Canada; ^4^ Dalhousie University Halifax NS Canada; ^5^ Nova Scotia Health Authority Halifax NS Canada; ^6^ North York General Hospital Toronto ON Canada; ^7^ Hamilton Health Sciences Hamilton ON Canada; ^8^ McMaster University Hamilton ON Canada; ^9^ Sunnybrook Health Sciences Centre Toronto ON Canada; ^10^ Michael Garron Hospital Toronto ON Canada; ^11^ IWK Health Centre Halifax NS Canada; ^12^ St. Michael's Hospital Toronto ON Canada

**Keywords:** healthcare workers, influenza, vaccine uptake

## Abstract

**Background:**

Influenza vaccine coverage rates among healthcare workers (HCWs) in acute care facilities in Canada remain below national targets.

**Objective:**

To determine factors associated with influenza vaccine uptake among HCWs.

**Methods:**

This secondary analysis of a prospective cohort study included HCWs aged 18‐69 years, working ≥20 h/wk in a Canadian acute care hospital. Questionnaires were administered to participants in the fall of the season of participation (2011/12‐2013/14) which captured demographic/household characteristics, medical histories, occupational, behavioural and risk factors for influenza. Generalized estimating equation logistic regression was used to determine factors associated with vaccine uptake in the season of participation.

**Results:**

The adjusted odds ratio for influenza vaccination in the current season was highest for those vaccinated in 3 of 3 previous seasons (OR 156; 95% CI 98, 248) followed by those vaccinated in 2 of 3 and 1 of 3 previous seasons when compared with those not vaccinated. Compared with nurses, physicians (OR 4.2; 95% CI 1.4, 13.2) and support services staff (OR 1.8; 95% CI 1.3, 2.4) had higher odds ratios for vaccine uptake. Conversely, HCWs identifying as Black had lower odds of uptake compared with those with European ancestry (OR 0.44, 95% CI 0.26‐0.75) when adjusted for other factors in the model.

**Conclusion:**

Healthcare workers differ in their annual uptake of influenza vaccine based on their past vaccination history, occupation and ethnicity. These findings indicate a need to determine whether there are other vaccine‐hesitant groups within healthcare settings and learn which approaches are successful in increasing their uptake of influenza vaccines.

## 1 INTRODUCTION

Influenza is responsible for 3‐5 million cases of severe illness and 250 000‐500 000 deaths each year worldwide.^1^ In healthcare settings, outbreaks of influenza can result in significant morbidity and mortality especially in those with compromised or immature immune systems including the elderly, children younger than 5 years of age and those with chronic underlying conditions.^2^, ^3^, ^4^

Despite seasonal fluctuations in vaccine effectiveness, influenza vaccines are considered an effective way to prevent disease and control transmission.^5^ The National Advisory Committee on Immunization and the World Health Organization recommend that individuals, including healthcare workers (HCWs), who may transmit influenza to those at higher risk of serious illness should receive the vaccine annually.^6^, ^7^ Even with studies showing that immunization of HCWs is associated with a decrease in nosocomial influenza illnesses and can reduce patient mortality,^8^, ^9^, ^10^ influenza vaccination coverage rates for HCWs in acute care facilities in Canada are well below the recommended national target of 80%.^11^

To maximize vaccination rates among the healthcare workforce, an understanding of which factors are associated with vaccine uptake is needed to help inform workplace immunization programs. This study's aim was to explore those factors for people working in Canadian acute care hospitals and who participated in a study of the risk factors associated with influenza illness.

## 2 METHODS

### 2.1 Data collection

Individuals were eligible to participate in the Influenza Cohort Study (ICS), which was conducted for four influenza seasons (2010/11 through 2013/14), if they were 18‐69 years old and working ≥20 h/wk in a participating acute care hospital (6 sites in Toronto, and starting in the 2011/12 season, 2 sites in Halifax and 2 sites in Hamilton). Each participant was followed for a single influenza season (approximately 1 November to 30 April). At enrolment, participants completed an online questionnaire detailing their medical history, influenza vaccination uptake in the previous 3 seasons, demographic characteristics, and occupational, community and behavioural risk factors for influenza. During the season, participants were asked to update their influenza vaccination status using an online form. Those with an unconfirmed vaccination status were contacted prior to the end of the season to confirm whether they had been vaccinated. For this analysis, participants were restricted to those participating in the 2011/12‐2013/14 seasons due to changes in the questionnaire from the previous season.

### 2.2 Study details

The outcome for this analysis was self‐reported influenza vaccination at any time during the season of study participation. The baseline questionnaire was used for demographic information and potential covariates. The selection of candidate variables associated with vaccine uptake was informed by a scoping literature review and included age, sex, physician diagnosed asthma, diabetes, hypertension, and heart disease, a history of cancer, influenza vaccination history for the three seasons prior to participation, previous laboratory (PCR)‐confirmed influenza illness, living with young children or older adults, occupation, work experience, ethnicity, physical activity, number of close patient contacts during a shift, and how frequently a surgical mask was worn when caring for patients with a febrile respiratory illness.

Occupational groups were categorized as nurses (nurse practitioners, registered, licensed practical and students), physicians (licensed, assistants and students), allied health (pharmacists, physiotherapists, respiratory therapists, psychologists, social workers, nutritionists, medical imaging technologists and students thereof) and support services (administration, patient attendants, housekeeping, ward coordinators, laboratory technologists, research and other support staff). The ethnic groups included European, East/South‐East Asian, South Asian/Middle Eastern, Black or other, which included Aboriginal, Latin American, West Indian, mixed and individuals reporting ethnicity as unknown. Physical activity was calculated using the International Physical Activity Questionnaire (IPAQ) scoring protocol and presented as a continuous variable.^12^

### 2.3 Statistical analysis

To account for participation in more than one season and to obtain population averaged estimates, we used generalized estimating equation (GEE) logistic regression analysis with an exchangeable correlation structure and conventional standard errors. Conventional standard errors were used due to the unbalanced design of the data. A backward selection procedure, removing variables with *P*‐values ≥ .20,^13^ was chosen to build multivariable models using variables listed in Table 1. Missing observations were excluded from the analyses and *P*‐values < .05 were considered statistically significant. Potential confounding variables (site and season of participation) were retained in all models regardless of their *P*‐value. Effect measure modification was assessed for sex by occupation through the addition of an interaction term and stratification of models. All models were assessed for fit, influential observations and over‐fitting. Data management and statistical analyses were performed using STATA Statistical Software.^14^

**Table 1:** Baseline characteristics, by season of study participation, 2011/12‐2013/14, Canada (n = 2436)

Factor (%)	2011/12 (n = 730)	2012/13 (n = 810)	2013/14 (n = 896)
Vaccinated, current season	562 (77.0)	618 (76.3)	654 (73.0)
Age, median (IQR)	42.7 (33, 51)	43.6 (34, 51)	44.3 (34, 52)
Sex			
Female	608 (83.3)	694 (85.7)	792 (88.4)
Male	122 (16.7)	116 (14.3)	104 (11.6)
Asthma	94 (12.9)	86 (10.6)	118 (13.2)
Diabetes	27 (3.7)	34 (4.2)	36 (4.0)
Heart disease	12 (1.7)	14 (1.7)	17 (1.9)
Hypertension	79 (10.8)	100 (12.4)	96 (10.7)
Cancer	6 (0.8)	9 (1.1)	6 (0.7)
Influenza vaccine history			
0/3	74 (10.2)	82 (10.1)	136 (15.2)
1/3	78 (10.7)	94 (11.6)	74 (8.3)
2/3	105 (14.4)	100 (12.4)	118 (13.2)
3/3	466 (63.9)	524 (64.7)	553 (61.7)
Unsure of ≥1 season	6 (0.8)	10 (1.2)	15 (1.6)
Influenza^a^, previous 3 seasons			
No	680 (93.2)	735 (90.7)	801 (89.4)
Yes	25 (3.4)	46 (5.7)	55 (6.1)
Don't know	25 (3.4)	29 (3.6)	40(4.5)
Child <5 y in home	115 (15.8)	112 (13.8)	129 (14.4)
Adult > 64 y in home	43 (5.9)	45 (5.6)	48 (5.4)
Occupation			
Nurse	297 (40.7)	298 (36.8)	344 (38.4)
Physician	42 (5.8)	46 (5.7)	35 (3.9)
Allied health	98 (13.4)	129 (15.9)	163 (18.2)
Support services	293 (40.1)	337 (41.6)	354 (39.5)
Years work experience (IQR)	12 (5, 23)	12 (5, 23)	13 (5, 25)
Close patient contacts (IQR)^b^	10 (4, 20)	10 (4, 20)	10 (4, 20)
Mask with FRI patient care			
No contact	288 (40.1)	334 (41.2)	359 (40.5)
Occasionally	77 (10.7)	81 (10.1)	77 (8.7)
Usually	78 (10.8)	88 (11.0)	83 (9.4)
Always	276 (38.4)	300 (37.7)	367 (41.4)
Ethnicity			
European	537 (73.6)	576 (71.2)	651 (72.3)
Black	42 (5.8)	50 (6.2)	44 (4.9)
East or South‐East Asia	75 (10.3)	92 (11.4)	96 (10.7)
S. Asia or Middle East	46 (6.3)	60 (7.4)	63 (7.0)
Other c	30 (4.1)	31 (3.8)	42 (4.7)
Physical activity, MET, min/wk (IQR)	2370 (834, 4158)	1866 (792, 3852)	1980 (895, 3816)

FRI: febrile respiratory illness; IPAQ: International Physical Activity Questionnaire; MET: metabolic equivalent; NC: not collected; S: south;

a Laboratory (PCR) confirmed influenza.

b Number of non‐household contacts within arm's reach and for 2 min or longer during a typical work day.

c Other: Aboriginal, Latin American, West Indian, mixed, not specified and unknown.

## 3 RESULTS

As seen in Table 1, there were 2436 participant‐seasons of observation over the 3 seasons of study. The median age was 43.5 years, 85.5% were female, and 38.5% were nurses. The overall influenza vaccination rate was 75.3%, and 63.3% of participants were vaccinated in all three seasons prior to participation.

In unadjusted analysis (Table 2), being vaccinated against influenza was significantly associated with previous influenza vaccination uptake, non‐nursing occupation, older age, more years of work experience, male, non‐Black ethnicity, and having diabetes mellitus or hypertension severe enough to require two or more visits per year with a healthcare practitioner.

**Table 2:** Factors associated with influenza vaccine uptake among healthcare workers in Canada

	Crude OR (95% CI)	*P*‐value	Adjusted OR^a^ (95% CI)	*P*‐value
Influenza vaccine history				
0/3	Referent		Referent	
1/3	5.32 (3.53, 8.02)	<.001	5.45 (3.40, 8.73)	<.001
2/3	22.9 (15.1, 34.9)	<.001	26.7 (16.7, 42.8)	<.001
3/3	126 (84.0, 188)	<.001	156 (98.4, 248)	<.001
Unsure of ≥1 season	4.55 (2.07, 10.0)	<.001	3.90 (1.68, 9.03)	.002
Occupation				
Nurse	Referent		Referent	
Physician	9.81 (3.98, 24.2)	<.001	4.24 (1.36, 13.2)	.013
Allied health	1.54 (1.16, 2.04)	.003	1.47 (0.99, 2.18)	.059
Support staff	1.33 (1.08, 1.63)	.006	1.79 (1.32, 2.42)	<.001
Years work experience	1.01 (1.00, 1.02)	.018		
Ethnicity				
European	Referent		Referent	
Black	0.46 (0.31, 0.68)	<.001	0.44 (0.26, 0.75)	.002
East or South‐East Asia	1.05 (0.75, 1.47)	.767	1.07 (0.69, 1.67)	.763
S, Asian or Middle East	0.89 (0.59, 1.32)	.565	1.60 (0.95, 2.71)	.078
Other b	0.74 (0.46, 1.18)	.211	0.55 (0.30, 1.02)	.060
Asthma (vs no)	1.48 (1.09, 1.99)	.010		
Age, y	1.01 (1.00, 1.02)	.001		
Male (vs female)	1.35 (1.01, 1.79)	.039		
Diabetes mellitus				
No	Referent			
Yes (0‐1 visits/y)	1.54 (0.68, 3.49)	.303		
Yes (2+ visits/y)	2.46 (1.16, 5.24)	.019		
Heart disease				
No	Referent			
Yes	1.49 (0.72, 3.09)	.275		
Hypertension				
No	Referent			
Yes (0‐1 visits/y)	1.19 (0.85, 1.66)	.318		
Yes (2+ visits/y)	1.84 (1.02, 3.31)	.042		
Cancer diagnosis (vs no)	1.41 (0.52, 3.81)	.499		
Influenza, previous 3 seasons				
No	Referent			
Yes	1.04 (0.74, 1.46)	.831		
Don't know	1.36 (0.91, 2.05)	.134		
Child < 5 y in home	0.97 (0.76, 1.24)	.806		
Adult > 64 y in home	0.89 (0.62, 1.29)	.541		
Close patient contact/d c	1.00 (0.99, 1.00)	.552		
Mask with FRI patient				
No patient contact	Referent			
Occasionally	1.05 (0.80, 1.38)	.709		
Usually	1.19 (0.89, 1.60)	.241		
Always	0.86 (0.71, 1.04)	.126		
Physical activity, MET min/wk	0.99 (0.99, 1.00)	.341		

FRI: febrile respiratory illness; HC: health care; IPAQ: International Physical Activity Questionnaire; IQR: interquartile range; MET: metabolic equivalent; S: South.

a Adjusted for season, site of participation and participation in >1 season.

b Aboriginal, Latin American, West Indian, mixed, not specified or unknown.

c Number of non‐household contacts within arm's reach and for 2 min or longer during a typical work day.

As shown in Table 2, factors associated with HCW vaccination uptake were previous vaccination history, occupation and ethnicity once the equation was adjusted for the impact of season, study site, and whether the individual participated in more than one season. Compared with HCW not vaccinated in any of the previous 3 seasons, the adjusted odds ratio of vaccine uptake increased stepwise from 5.4 (95% CI 3.4, 8.7) for those vaccinated once in the 3 previous seasons to 156 (95% CI 98, 248) for those vaccinated in all 3 of the 3 previous seasons. Physicians (OR = 4.2, 95% CI 1.4, 13.2) and hospital support services staff (OR = 1.8, 95% CI 1.3, 2.4) had significantly higher odds of vaccine uptake in the current season than nurses. In addition, participants identifying as Black had lower adjusted odds ratios for vaccine uptake in the current season than HCW of European ancestry (OR = 0.44, 95% CI 0.26‐0.75). There were no significant differences in vaccine uptake for participants of other ethnic backgrounds. A sensitivity analysis excluding influenza vaccine history produced a similar model with substantially similar estimates.

## 4 DISCUSSION

In this multiseason study of adults working in acute care hospitals in Canada, the odds ratio for influenza vaccine uptake increased exponentially with every additional year of influenza vaccine uptake over the previous three seasons. Similar findings have been illustrated in studies from the USA, Europe and China,^1^, ^15^, ^16^, ^17^, ^18^, ^19^, ^20^, ^21^ suggesting that the individual perceptions associated with vaccine acceptance and refusal remain stable over several years. It is likely necessary to tailor immunization programs differently to reach HCW at all levels of uptake, from refusers to those accepting influenza vaccine intermittently to those vaccinated annually. Reviews of the literature report that believing that influenza vaccine is effective, that influenza is highly contagious, and that prevention is important (including HCWs’ roles in transmission) are predictive of influenza vaccination.^22^, ^23^, ^24^ Further research is needed to determine how common concerns related to vaccine hesitancy in HCW (e.g vaccine safety and efficacy, perceived low risk of illness)^25^, ^26^ are associated with the frequency of vaccine uptake in HCW working in settings with different vaccination policies and work cultures.

Another finding in our study illustrated that nurses, the largest occupational group within acute care settings, were less likely to be vaccinated than other occupational groups. Our data are consistent with findings from several other studies in that vaccine uptake is significantly lower for nurses than physicians.^18^, ^27^, ^28^, ^29^, ^30^, ^31^, ^32^, ^33^ One study determined that physicians had a higher level of knowledge about influenza and influenza vaccines were less likely to expect a severe reaction to the vaccine, and more likely to consider influenza vaccines effective than nurses.^18^ Similarly, increased knowledge levels and beliefs that influenza is a serious illness and that vaccinations are safe were associated with higher uptake among nurses.^31^ Although targeted educational strategies may be needed to resolve misconceptions^22^, immunization programs focusing solely on education result in only minimal increases in coverage rates.^34^, ^35^ Evaluations of programs targeting nurses suggest that multipronged approaches are necessary^36^, ^37^, ^38^, ^39^ to address the myriad reasons for vaccine hesitancy and refusal in this occupational group.^24^, ^40^

Lastly, in our study population, people identifying as Black had lower odds of being vaccinated against influenza compared with those with European heritage. Earlier findings of ethnicity's association with vaccine uptake have been contradictory. One US study reported no difference in pandemic influenza A(H1N1) vaccine uptake by ethnicity^41^ while another study reported lower rates of vaccination in non‐Hispanic Black than non‐Hispanic White HCWs.^42^ Higher levels of concern about vaccine safety and effectiveness coupled with lower concern about the seriousness of influenza disease were found to mediate this finding.^42^

Limitations of our study include volunteer bias, with a higher uptake of the influenza vaccine in our participants than reported for HCWs in Canada.^11^ It is possible that some participants incorrectly recalled their vaccination history, especially if they received the influenza vaccine intermittently. However, they were able to answer “do not remember” to the question and other studies have found that self‐report of influenza vaccination status is reliable and valid.^43^, ^44^

The main strengths of the study were its sample size (over 2400 person‐years) with robust estimates produced by the model. The modelling approaches explored effect measure modification and confounding, which were applied consistently across models and data were collected from multiple sites, three cities and over three seasons allowing for a more representative sample and generalizable findings.

As expected, HCWs who routinely receive their influenza vaccine are significantly more likely to be vaccinated again in the current season. Likewise, the odds of being vaccinated drop precipitously for every season of non‐vaccination in their recent history. In our sample, nurses were the occupational group least likely to be vaccinated and people identifying as Black were the ethnic group least likely to be vaccinated against influenza. These findings may prove useful by immunization campaign teams who are able to tailor strategies to reach specific groups.

## CONFLICT OF INTERESTS

None.
